# The current physical activity in persons with disability in Qatar: a cross-sectional study

**DOI:** 10.3389/fspor.2024.1394097

**Published:** 2024-12-03

**Authors:** Uma Pandiyan, Ibin Kariyathankavil, Abderrahmane Rebbouh, Leila Khairallah EP Grami, Asha Susan Thomas, Brijesh Sathian

**Affiliations:** ^1^ Department of Physical Medicine & Rehabilitation, Hamad Medical Corporation, Doha, Qatar; ^2^ Department of Physiotherapy, Hamad Medical Corporation, Doha, Qatar; ^3^ Geriatrics and Long-Term Care Department, Rumailah Hospital, Hamad Medical Corporation, Doha, Qatar

**Keywords:** persons with disability, physical activity, psychomotor impairment, physical impairment, sports, Qatar

## Abstract

**Purpose:**

The primary objective of this study was to measure and quantify the current level of physical activity among persons with disability in Qatar. The secondary objective was to assess and analyze the duration of sedentary behavior among individuals with disability in Qatar.

**Methods:**

This was a cross-sectional epidemiological study of persons with disability living in Qatar. The study period was from October 2020 to December 2021

**Results:**

96 persons with disabilities participated in this study. They included individuals with amputations, spinal cord injuries, cerebral palsy, and other developmental impairments. Of the respondents, 56% were male and 64% were Qataris. Of the respondents, 61.5%said that they felt psychologically and emotionally better when engaging in physical activity, and this difference was statistically significant. 71% of all those who reported feeling physically good after sport, 65% said that they enjoyed the social aspects of sports. This corroborates the beneficial effects of exercise, sports, and physical activity in persons with all types of disabilities.

**Conclusion:**

There was a definite impact on the emotional and physical well-being of those who participated in the physical activities. There was less participation in team activities and two-thirds preferred to exercise alone. However, their numbers were not statistically significant because of low enrollment in the study.

## 1 Introduction

Physical activity refers to the movement of the body produced by the musculoskeletal system that requires energy expenditure. Regular physical activity promotes normal growth and development and can make individuals feel better, perform better, and improve sleep pattern (1). Studies have shown that physically active individuals enjoy better mental health and a decrease in non-communicable diseases, such as diabetes, hypertension, and coronary artery disease, as well as a decrease in cancer risk and a better quality of life. Obesity and overweight have been proven to lead to the above-mentioned diseases (2). There is inadequate literature related to physical activity in persons with disabilities in Qatar. These barriers can be either intrinsic or extrinsic.

Group activities and team sports teach children the skills required to live in a community. The United Nations Disability Report 2021 states that there are 240 million children with disabilities in the world: 34% are stunted, 25% are likely to be wasted, 41% feel discriminated against, and 51% feel unhappy (3).

Exercise improves sleep and reduces the risk of falls in older adults by improving balance and joint mobility. It prevents sarcopenia and osteoporosis and delays cognitive impairment and dementia. This improves the quality of life. Information on the benefits of physical activity is well known across age groups, but data on physical activity in persons with disabilities are deficient in Qatar. Therefore, we initially reviewed the literature on the trends of overall lifestyle and physical activity in this region and subsequently focused on persons with disabilities in Qatar.

In the Middle East, climatic, social, and cultural practices make physical activity a challenge. The high temperatures in arid environments make outdoor activities challenging for about six months. Citizens must depend on indoor physical activities to stay healthy. Expatriates form a large part of the blue- and white-collar working populations. Due to the diversity and shifting population there are layers of inclusion in living and working spaces. These indoor sports facilities are gender specific and not always accessible to the entire population. Adele Khodr, the Regional Director of UNICEF – MENA (Middle East North Africa) region in her foreword on the Disability report remarks about the stigma of children with disabilities being isolated and excluded from society and their communities (4).

The World Health Survey, in collaboration with the World Health Organization (WHO), conducted a survey in Qatar in 2006. It revealed that 32.7% of adults were overweight and 28.7% were obese. It also indicated that 28.7% of children under five years of age were overweight. This was ascribed to the population's changing lifestyle in relation to nutrition and physical activity (5, 6). The survey revealed that 56% of the population did not receive the physical activity levels recommended by the WHO (at least 30 min of moderate physical activity five days a week). Fourteen percent of the population were diagnosed with hypertension and required medication. Hyperlipidemia was observed in 27% of women and 24% of men living in Qatar. If these comorbidities are not addressed they could lead to disabilities in the future. Establishing a comprehensive plan to control chronic non-communicable disease factors was crucial. The increasing challenge of obesity and its associated diseases has led the Supreme Council of Health to formulate the Qatar National Nutrition and Physical Activity Action Plan (2011–2016). This Plan included a comprehensive nutrition and physical activity program with initiatives targeted at various stakeholders to reduce morbidity and mortality due to chronic non-communicable diseases (7). Media awareness programs are encouraged to promote healthy choices. The stakeholders and management of this plan included the Supreme Council of Health, Supreme Education Council, Hamad Medical Corporation, Ministry of Health and Labor, Ministry of Municipality and Agriculture, and Business and Trade. The Qatar National Physical Activity Guidelines 2014 – provided details about the exercise requirements of different age groups, gender (pregnancy), Ramadan, and during summer (8).

In 2006, the United Nations Convention on the Rights of Persons with Disabilities (UN CRPD) stated that social and community inclusion for persons with disabilities in well-resourced and resource-scarce settings depends on access to mobility aids, assistive technologies, accessible environments, and opportunities for physical activity (9). The rights to which all people are entitled include full participation in the economic, educational, health, social, and recreational dimensions of society. Qatar was one of the 162 signatories in this declaration. It is a move to shift the focus on persons with disabilities as objects of charity requiring social protection and medical management, towards considering them as full and equal members of society with human rights. It promotes human dignity. The term disability sport includes all sport and physical activity for leisure and recreation by persons with disability including para sports (10). Participation in disability-adapted sports or parasports is increasing internationally in developed countries. Qatar has been participating in the Paralympics since 1996 and has 232 athletes registered in the Qatar Paralympic Committee (QPC) (10). However, its implementation remains challenging. There were only two participants from Qatar in the Paris 2024 Summer Paralympics. An improved understanding of why some persons with disabilities are more physically active and others are unable to be active needs to be comprehensively addressed.

The primary objective of this study was to measure and quantify the current level of physical activity among individuals with disabilities in Qatar.

The secondary objective was to assess and analyze the duration of sedentary behaviors among individuals with disabilities in Qatar.

## 2 Methods

### 2.1 Study design and participants

This was a cross-sectional study involving persons with disabilities living in a community in Qatar. The UN CRPD definition for people with disabilities was used (11). Individuals with established disabilities (mobility, intellectual and developmental delays, hearing impairment, and visual impairment) participated in the study. Those with mental instability, recent seizures, breathing difficulties, acute bone and joint injuries, and those who were bed-bound (chronic vegetative state) were excluded from the study. Mental disorders (anxiety, paranoid behavior) were excluded because they would cause more distress. An online questionnaire in Arabic and English with 16 questions was shared by the research team members after informed consent was obtained at the onset of the survey. The age group included was–10–70 years of age. The subjects included had to be medically stable and had to have the ability to understand and communicate and provide consent. The decision to include 10–18 was taken, as there is a significant number of individuals with disabilities in this age group (12). In situations where an individual was less than 18 years of age, the parents gave consent and provided information.

Qatar does not have a disability register yet. The participants of the study were recruited by those attending the outpatient clinic in Qatar Rehabilitation Institute and via email and WhatsApp to individuals with visual, hearing, and movement limitations through their Associations, and the Paralympic Association of Qatar. (The Visual and Hearing-impaired Associations had contact numbers of their clients) Responses from persons with autism, cerebral palsy, and intellectual impairment who could not respond or use devices to record responses were provided by their parents.

Convenience method and snowball sampling was used. Data were collected by the primary investigator using Google sheets and stored in a password-protected electronic format.

The study period was October 2020 to December 2021. The sample size was 96 persons with disabilities (300 persons as per the original plan).

### 2.2 Outcome measures

The questionnaire collected demographic data, type of impairment, frequency, duration, and impact of physical activity in persons with disabilities. The questionnaire was initially developed by the ISPRM (International Society of Physical and Rehabilitation Medicine) Task Force after a literature review of stakeholder perspectives on people with disabilities. There were three sections to the survey. Section 1 of the questionnaire collected the demographic data. Section 2 of the survey was adapted from Parts 4 and 5 (recreation, sports and leisure time physical activity, and time spent sitting) of the International Physical Activity Questionnaire (IPAQ). IPAQ's Part 1–3 deal with job, related, transportation related and household activity and hence were excluded. Section 3 provides information on the emotional impact of PA (physical activity). The multiple-choice format of answers was designed and administered on Google Forms and collected on Google sheets.

The questions were adapted to the sociocultural demographics of people living in Qatar (12). Qatar has less than 12% Qataris and 88% expatriates. In Section 1 of our questionnaire, we collected data on the nationalities of six countries (Indian, Filipino, Jordanian, Algerian, Bangladeshi, Nepali, and others). The gender ratio is skewed with 77% males and 23% females. Only medically fit expatriates are permitted to work. Accessibility challenges exist due to gender, occupation, income, and socioeconomic factors.

Kathleen A Martin Ginis et al's study in the Lancet regarding measurement of physical activity in persons with disability with IPAQ have little validity and reliability data for population with disability. People with disabilities are completely inactive, and efforts to transition to low levels of activity could have a major positive effect (13). Recent research has shown that physical activity in individuals with neurological impairments causes a physiological response and attenuates the cardiometabolic response. Similarly, those using ambulatory aids and wheelchairs did not have a homogenous response; hence, categorical scoring of the level of physical activity of IPAQ into low, moderate, and high used for persons without disability was not used (14).

### 2.3 Statistical analysis

Descriptive statistics were used to summarize the demographic characteristics of the respondents. Normally distributed data and results are reported as mean and standard deviations (SD); the other results are reported as median and interquartile ranges (IQR). Categorical data were summarized as frequencies and percentages. The focus of the statistical data analysis in this study was to determine and assess the present level of physical activity in individuals with disabilities and associated factors. Associations between two or more qualitative variables will be assessed using the chi-square (*χ*^2^) or Fisher's exact tests, as appropriate. Statistical significance was set at *p* < 0.05. All Statistical analyses were performed using R.4.4.1 version.

## 3 Results

### 3.1 Socio-demographic characteristics

Table 1 shows that a total of 96 participants were included in this study. The majority were between 10 and 34 years of age (66.7%, *n* = 64), followed by those aged 35 to 49 (25%, *n* = 24), and those aged 50 and above (8.3%, *n* = 8). Male participants comprised 56.3% (*n* = 54), while females constituted 42.7% (*n* = 41). (one participant did not answer this question) Most participants were Qatari nationals (63.5%, *n* = 61), with non-Qataris accounting for 36.5% (*n* = 35). Regarding impairment types, psychomotor impairment was most prevalent (42.7%, *n* = 41), followed by other impairments (hearing and visual, 35.4%, *n* = 34) and physical impairments (21.9%, *n* = 21).

**Table 1:** Socio demographic information.

Variable	Category	Count
Age	10–34	64 (66.7%)
	35–49	24 (25%)
	50 above	8 (8.3%)
Gender	Male	54 (56.3%)
	Female	41 (42.7%)
Nationality	Qatari	61 (63.5%)
	Non Qatari	35 (36.5%)
What is your impairment?	Physical impairment	21 (21.9%)
	Psychomotor	41 (42.7%)
	Other (hearing and visual)	34 (35.4%)

### 3.2 Physical activity patterns

Table 2 depicts that most participants reported sitting for more than 3 h on weekdays (53.1%, *n* = 51) and weekends (54.2%, *n* = 52). Approximately 38.5% (*n* = 37) of participants walked for at least 10 min on 3–5 days in the past week, while 14.6% (*n* = 14) were not ambulatory. For those who walked, 33.3% (*n* = 32) did so for more than 30 min. The most commonly reported sports activities were cycling (18.8%, *n* = 18) and special football (17.7%, *n* = 17), with 32.3% (*n* = 31) engaging in other activities such as walking.

**Table 2:** Details of physical activity.

Variable	Category	Count
During the last 7 days, how much time did you usually spend sitting on a weekday?	<1 h	7 (7.3%)
	1–3 h	20 (20.8%)
	>3 h	51 (53.1%)
During the last 7 days, how much time did you usually spend sitting on a weekend?	<1 h	5 (5.2%)
	1–3 h	19 (19.8%)
	>3 h	52 (54.2%)
During the last 7 days, on how many days did you walk for at least 10 min at a time in your leisure time?	<2 days	20 (20.8%)
	3–5 days	37 (38.5%)
	>5 days	25 (26.0%)
	Not ambulatory	14 (14.6%)
How much time did you spend on those days walking in your leisure time?	<15 min	26 (27.1%)
	16–30 mins	26 (27.1%)
	>30 min	32 (33.3%)
What sport(s) did you play?	Bicycling	18 (18.8%)
	Goal ball	2 (2.1%)
	Special football	17 (17.7%)
	Athletics	8 (8.3%)
	Others	31 (32.3%)
Think about only those physical activities that you did for at least 10 min at a time. During the last 7 days,	<2 days	21 (21.9%)
	3–5 days	35 (36.5%)
	>5 days	13 (13.5%)
How much time did you usually spend on one of those days doing moderate to vigorous physical activities in your leisure time?	<30 Min	19 (19.8%)
	30–1 h	34 (35.4%)
	>1 h	20 (20.8%)
How many people did you play with at one time?	0 (alone)	30 (31.3%)
	1–2 persons	23 (24.0%)
	3 or more	19 (19.8%)
	NA	24 (25.0%)
I generally feel psychologically and emotionally better when I regularly exercise compared to when I do not exercise.	Strongly agree/agree some what	59 (61.5%)
	Neither agree or disagree	25 (26.0%)
	Disagree somewhat/strongly disagree	12 (12.5%)
I generally feel physically good when I regularly exercise and play sports compared to when I do not	Strongly agree/agree some what	68 (70.8%)
	Neither agree nor disagree	13 (13.5%)
	Disagree somewhat/strongly disagree	15 (15.6%)
I enjoy the social aspects of exercise and sports	Strongly agree/agree some what	63 (65.6%)
	Neither agree nor disagree	20 (20.8%)
	Disagree somewhat/strongly disagree	13 (13.5%)

### 3.3 Psychological and emotional impact of exercise

Participants generally agreed that regular exercise had a positive impact on their psychological and emotional well-being (61.5%, *n* = 59), and 70.8% (*n* = 68) felt physically better when exercising regularly. Furthermore, 65.6% (*n* = 63) enjoyed the social aspects of exercise and sports.

### 3.4 Association between impairment types and socio-demographic variables

Table 3 shows that cross-tabulation analysis revealed significant differences between impairment types and various socio-demographic factors. For nationality, Qatari participants had a higher prevalence of physical impairment (81.0%) and psychomotor impairment (75.6%) compared to hearing and visual impairments, where non-Qataris were more represented (61.8%; *p* = 0.001). Males were significantly more likely to have psychomotor impairment (82.5%, *p* = 0.001). Regarding age, psychomotor impairment was more prevalent in the 10–34 age group (78.0%, *p* = 0.009).

**Table 3:** Cross tabulation of physical impairment, psychomotor, hearing and visual with socio demographic and other variables.

Variable	Category	Physical impairment	Psychomotor	Other (hearing and visual)	*P*-value
Nationality	Qatari	17 (81.0%)	31 (75.6%)	13 (38.2%)	0.001
	Non-Qatari	4 (19.0%)	10 (24.4%)	21 (61.8%)	
Gender	Male	9 (42.9%)	33 (82.5%)	12 (35.3%)	0.001
	Female	12 (57.1%)	7 (17.5%)	22 (64.7%)	
Age group	10–34	13 (61.9%)	32 (78.0%)	19 (55.9%)	0.009
	35–49	8 (38.1%)	8 (19.5%)	8 (23.5%)	
	50 above	0 (0.0%)	1 (2.4%)	7 (20.6%)	
Do you exercise or not?	Yes	20 (95.2%)	38 (92.7%)	24 (70.6%)	0.009
	No	1 (4.8%)	3 (7.3%)	10 (29.4%)	
Where do you exercise and/or play sports	Home	7 (35%)	16 (42.1%)	6 (25%)	0.013
	Community gym or recreation center	1 (5%)	16 (42.1%)	9 (37.5%)	
	Outpatient hospital setting	5 (25%)	1 (2.6%)	4 (16.7%)	
	Other	7 (35%)	5 (13.2%)	5 (20.8%)	
During the last 7 days, how much time did you usually spend sitting on a weekday	<1 h	3 (21.4%)	0 (0.0%)	4 (13.3%)	0.013
	1–3 h	4 (28.6%)	6 (17.6%)	10 (33.3%)	
	>3 h	7 (50.0%)	28 (82.4%)	16 (53.3%)	
During the last 7 days, how much time did you usually spend sitting on a weekend?	<1 h	3 (23.1%)	0 (0.0%)	2 (6.7%)	0.001
	1–3 h	0 (0.0%)	6 (18.2%)	13 (43.3%)	
	>3 h	10 (76.9%)	27 (81.8%)	15 (50.0%)	
During the last 7 days, on how many days did you walk for at least 10 min at a time in your leisure time?	<2 days	6 (28.6%)	6 (14.6%)	8 (23.5%)	0.237
	3–5 days	5 (23.8%)	22 (53.7%)	10 (29.4%)	
	>5 days	7 (33.3%)	9 (22.0%)	9 (26.5%)	
	Not ambulatory	3 (14.3%)	4 (9.8%)	7 (20.6%)	
How much time did you spend on those days walking in your leisure time?	<15 mts	10 (55.6%)	7 (18.9%)	9 (31.0%)	0.022
	16–30 mts	4 (22.2%)	10 (27.0%)	12 (41.4%)	
	>30 mts	4 (22.2%)	20 (54.1%)	8 (27.6%)	
What sport(s) did you play?	Bicycling	9 (52.9%)	8 (22.2%)	1 (4.3%)	0.001
	Goal ball	0 (0.0%)	1 (2.8%)	1 (4.3%)	
	Special Football	0 (0.0%)	15 (41.7%)	2 (8.7%)	
	Athletics	2 (11.8%)	3 (8.3%)	3 (13.0%)	
	Others (walking)	6 (35.3%)	9 (25.0%	16 (69.6%)	
Think about only those physical activities that you did for at least 10 min at a time. During the last 7 days	<2 days	4 (33.3%)	7 (19.4%)	10 (47.6%)	0.003
	3–5 days	3 (25.0%)	26 (72.2%)	6 (28.6%)	
	>5 days	5 (41.7%)	3 (8.3%)	5 (23.8%)	
How much time did you usually spend on one of those days doing moderate to vigorous physical activities in your leisure time?	<30 min	8 (53.3%)	6 (18.8%)	5 (19.2%)	0.013
	30–1 h	4 (26.7%)	13 (40.6%)	17 (65.4%)	
	>1 h	3 (20.0%)	13 (40.6%)	4 (15.4%)	
How many people did you play with at one time?	0 (alone)	10 (47.6%)	9 (22.0%)	11 (32.4%)	0.307
	1–2 Persons	4 (19.0%)	11 (26.8%)	8 (23.5%)	
	3 or more	2 (9.5%)	12 (29.3%)	5 (14.7%)	
	Not applicable	5 (23.8%)	9 (22.0%)	10 (29.4%)	
I generally feel psychologically and emotionally better when I regularly exercise compared to when I do not exercise	Strongly agree and agree some what	10 (47.6%)	29 (70.7%)	20 (58.8%)	0.311
	Neither agree or disagree	6 (28.6%)	9 (22.0%)	10 (29.4%)	
	Disagree somewhat or strongly disagree	5 (23.8%)	3 (7.3%)	4 (11.8%)	
I generally feel physically good when I regularly exercise and play sports compared to when I do not	Strongly agree and agree some what	13 (61.9%)	33 (80.5%)	22 (64.7%)	0.278
	Neither agree or disagree	4 (19.0%)	5 (12.2%)	4 (11.8%)	
	Disagree somewhat or strongly disagree	4 (19.0%)	3 (7.3%)	8 (23.5%)	
I enjoy the social aspects of exercise and sports	Strongly agree and agree some what	15 (71.4%)	29 (70.7%)	19 (55.9%)	0.160
	Neither agree or disagree	3 (14.3%)	10 (24.4%)	7 (20.6%)	
	Disagree somewhat or strongly disagree	3 (14.3%)	2 (4.9%)	8 (23.5%)	

### 3.5 Exercise habits across impairment types

Participants with psychomotor impairment were more likely to exercise regularly (92.7%, *p* = 0.009), compared to those with hearing and visual impairments (70.6%). Regarding exercise location, individuals with physical impairment predominantly exercised at home (35%), while those with psychomotor impairment favored community gyms (42.1%, *p* = 0.013).

### 3.6 Sedentary behavior and impairment type

Those with psychomotor impairments were more likely to spend over 3 h sitting on both weekdays (82.4%, *p* = 0.013) and weekends (81.8%, *p* = 0.001), compared to those with other impairments.

### 3.7 Sports participation and duration of physical activity

Bicycling was more common among participants with physical impairments (52.9%, *p* = 0.001), while special football was predominant among those with psychomotor impairments (41.7%). Walking was the most common activity among those with hearing and visual impairments (69.6%).

### 3.8 Psychosocial aspects of exercise

There were no statistically significant differences between impairment types in terms of the psychological and social benefits of exercise. However, those with psychomotor impairment were most likely to strongly agree or agree that they felt better physically when exercising (80.5%, *p* = 0.278).

In summary, significant differences were observed in physical activity patterns and preferences across different impairment types, highlighting the need for tailored interventions to promote physical activity within this population.

## 4 Discussion

### 4.1 Microlevel – study related

This study was originally conceived by the ISPRM Task Force on Physical Activity in Persons with Disabilities. They initially planned a multicentric survey to assess global situation on the existing level of Physical Activity in persons with disabilities living in the community in late 2019. However, with the onset of the COVID - 19 pandemic, the study was replaced by another online survey to assess the impact of the pandemic on people with disabilities (15). The Task Force member from Qatar had submitted the proposal to undertake the study to Hamad Medical Corporation Institution Review Board, and this study ensued. The aim of this study was to assess the existing physical activity of persons with disabilities living in the community in Qatar. The questionnaire was validated through a pilot survey.

The prevalence of disability in Qatar is 0.19% (16). 18,360 persons with disability as per the 2020 census. The participation rate could not be definitively ascertained as it was an online survey. This survey collected demographic information about persons with disabilities living in the community, the frequency of physical activity, and the details of the activity, where and with whom they participated in the physical activity. The participation in this study was one third of the sample size planned. There could have been a stigma toward sharing information about their disabilities online. Illiteracy, incomplete comprehension of these queries could be another reason. Digital barriers could have been another reason, as they required the ability to use smartphones or tablets and a stable Internet connection. The COVID-19 pandemic and lockdown was another hurdle in accessing individuals with disabilities for this study.

There was no access to adapted sports as an option of therapy at the primary venue (Qatar Rehabilitation Institute) of the study. The individuals who participated in shotput, javelin, discus and powerlifting were trained at the Qatar Paralympic committee.

### 4.2 Mid level – Qatar – Middle East

In our study in Qatar, we found that 61.5% of persons with disabilities felt psychologically and emotionally better with regular physical activity, and 65.6% enjoyed the social aspect of sports. The inclusive 22nd FIFA Football World Cup played a transformative role in making the entire country accessible to persons with disabilities (17). The challenge now is to sustain change and promote inclusive and adaptive options to promote well-being in persons with disabilities.

The Accessibility Report of the World Innovative Summit on Health (WISH 2022) addressed the building of a legacy of inclusion. The Report stated that socially constructed obstacles create challenges for people with disabilities and that removing them would create equality and offer people with disabilities more independence and control over their lives (18). These challenges could be physical, they could be due to attitudes or information and communication, barriers.

In our study, we found that 54% of people with disabilities were sedentary on weekends, and more than half of them reported being sedentary on weekdays. Less than a quarter of the study population participated in team or group activities. There was a gender disparity among those who participated in group activities and indoor and outdoor activities. This was similar to the observations of Al-Thani et al. about considerable gaps in the lifestyles of individuals living in Qatar. He found that 70% of adults were overweight or obese (19). Rahim et al. found that in the Arab world development, mechanization, and urbanization, had impacts on the diet, physical activity, and body composition of individuals living there and the governments have not yet implemented policies to reduce Noncommunicable diseases (20). This could involve human and economic burdens on people with and without disabilities.

From a sociological perspective the physical activity of persons with disabilities can be examined within the context of the state of affairs in Qatar over the last five years. Qatar experienced a blockade for approximately four years, superadded by the COVID -19 pandemic and then the run-up for the Football World Cup 2022. Physical Activity for sports and exercise belongs to the middle and top of Maslow's pyramid of hierarchy of needs; it is a psychological or self-fulfillment need (21). The Qatar blockade, and COVID -19 pandemic disrupted the basic needs of biological, physiological, financial, and relationships with family and friends. Physiological and Safety needs: the base of the pyramid was impacted. The Doha International Family Institute (Qatar Foundation) report (22) offers insights into the critical impact of the sudden blockade on families and individual well-being. It describes family fragmentation, abuse via social media, psychological features of anxiety, fear, depression, bullying, marital life, financial stress, and gaps in service.

To the credit of Qatar, there is an awareness of the inclusion of persons with disabilities in its national policies Qatar National Vision 2030 (23). Aspetar, a premier orthopedic and sports hospital, has updated the Qatar National Physical Activity Guidelines in 2021 (24). The inclusion of persons with disability in sport – rights and challenges and best practices in sport have been published by the British journal of sports medicine in 2022 too (25). In 2022 the editorial in British Journal of Sports Medicine highlighted Qatar Foundation Ability Friendly program (AFP). This opportunity is not available to all residents of Qatar due to logistical issues (26). Our study confirms a gap in actual physical activity outcomes in the community despite these guidelines. There is a need to promote the Ability Friendly Program across Qatar and the Middle East and a demand to train competent coaches to coach children with disabilities.

The WHO Integrated Care for Older People (ICOPE) screening tool in Qatar addresses older individuals with chronic health issues or disabilities at primary, secondary and tertiary levels (27). It needs to translate to improved physical activity and healthy ageing.

The report of the United Nations Economic and Social Commission for West Asia on Arab cities suggests the need for a cohesive strategic vision that requires a representative process that includes males, females, youth, ethnic and religious groups, the elderly, persons with disabilities, resettled migrants, refugees, the unemployed, businesses, NGO's, etc. (28). The United Nations Habitat the State of Arab Cities report 2022 shows the cleft in the Arab World and the extent to which this negatively affects sociocultural sustainability, among other things (29). He observed a setting where one can perceive the stress of modernity and tradition, religiosity and secularism, exhibitionism, and veiling. He deems it a place of contradiction and paradox. Each of these portrayals plays into a drab form of an Arab or Middle Eastern city. Gymnasiums are gender specific. Certain Parks and beaches are not permissible for men without families. When we examine the daily physical activity of persons with disabilities in this context, we can see gaps in accessibility. A multilevel approach in the Arab region, at the national and local levels, is required to provide a framework to improve physical activity in this group.

### 4.3 Macro level

People with disabilities differ in their physical activity depending on the type and severity of their disabilities. It also depends on intrapersonal, interpersonal, and structural imbalances (30). Intrapersonal can vary according to individual preferences, the availability of assistive devices, mobility aids, and accessibility to public spaces. Interpersonal could depend on their caregivers or the behavior patterning of interpersonal relationships (31). The cotton-wool parenting approach for children with disabilities could prevent them from growing and exploring physical activities. In their survey of youth with disabilities in Sport England, Finch et al. found that they were unlikely to participate in extracurricular or outside school sports activities due to a lack of transport and funding to clubs (32). Kung et al. in their analysis of use of public sports facilities in persons with disability showed that women and individuals older than 45 participated more than the male counterparts. They also found that sports participation rates decreased as individuals grew older. Another barrier to participation in sports is that they depend on others (33).

The ecological model of physical activity in persons with disability addresses the multiple layers which influence decisions to be physically active across the lifespan from individual factors to interpersonal, environmental and global (34, 35). It is relevant to note that extreme weather conditions and natural and man-made disasters (including war) affect physical activity in this vulnerable population.

Our study observed the physical activity of persons with disabilities in this scenario. The data reflect the resilience of the respondents and their families. It is worth reconsidering that physical activity concurrently improves personal, environmental, and social competencies in a rapidly changing world. As healthcare professionals, it is our desire to enable clients to transcend barriers from an individual level to the community level by anticipating and addressing appropriate organizational policies.

## 5 Strengths and limitations

This study was an online survey; hence, it was easy to access people with disabilities in the community. It provides data on the current physical activity of individuals with disabilities in Qatar, in line with the second pillar of social development, Qatar National Vision 2030.

This study has several limitations. The survey was online and had limited potential for obtaining all details of disability. Hence, it was simplified to movement or physical impairment, psychomotor including cognitive, and others (visual, hearing), and there could have been a reporting bias. However, the response rate was low. This study was a one-time analysis and did not evaluate changes with interventions. This study did not address the physical activity of the targeted age groups of children and adolescents, adults, and elderly individuals. The Participation rate could not be ascertained as it was an online survey.

In conclusion, participation in physical activity had a favorable impact on emotional and physical well-being in persons with disabilities. Future plans of the Qatar Foundation's Ability Friendly program to collaborate with stakeholders need to be explored (36). There is a requirement for anti-stigma awareness programmes Qatar has policies, plans, and investments to involve physical activity in persons with disabilities. However, the operationalizing of the National Vision requires consistent collaboration at all levels to include persons with disability.

## Data Availability

The original contributions presented in the study are included in the article, further inquiries can be directed to the corresponding author.
